# Supplementary material for: An integrated IKOA-CNN-BiGRU-Attention framework with SHAP explainability for high-precision debris flow hazard prediction in the Nujiang river basin, China
Source: PLoS One. 2025 Jun 24;20(6):e0326587. doi: 10.1371/journal.pone.0326587 (PMC12186985; doi:10.1371/journal.pone.0326587)
Supplement: Attachment — Submitted filename: Response to Reviewers.docx [file pone.0326587.s002.docx]

**Response to Editor and Reviewers**

Dear editors and reviewers,

Thank you for your letter and for the reviewers' comments concerning our manuscript entitled “An Integrated IKOA-CNN-BiGRU-Attention Framework with SHAP Explainability for High-Precision Debris Flow Hazard Prediction in the Nujiang River Basin, China” (ID: PONE-D-25-14786). Those comments are all valuable and very helpful for revising and improving our paper, as well as providing important guiding significance to our research. We have studied comments carefully and have made corrections which we hope meet with approval. We also highlighted the changes using red fonts in the attached Revised full manuscript file to make it easier for you to check our revision. The principal revisions made within this paper and responses to the reviewers' comments are delineated as follows.

Sincerely Yours,

Dr. Tianlong Wang

**Response to Reviewer 1**

(Original comments and queries are in blue, while our responses are in black)

**[General Comment]**

This manuscript presents an integrated framework that combines IKOA-CNN-BiGRU-Attention architecture with SHAP explainability analysis for debris flow hazard prediction in the Nujiang River Basin, China. The research demonstrates innovation in the field of debris flow hazard prediction, particularly in the integration of optimization algorithms, deep learning architectures, and explainability mechanisms. However, some methodological issues should be addressed, particularly regarding the validation of prediction accuracy, reconciliation of variable importance rankings, and justification of analytical choices.

**Response:**

We thank Reviewer 1 for their recognition of the manuscript’s methodological innovation and for providing nine detailed and constructive comments, which have been instrumental in refining the quality and clarity of our work. In this revision, we have systematically addressed each of the reviewer’s specific points, and all corresponding modifications have been carefully incorporated and are clearly marked in red in the revised manuscript. In particular, Section 6 has been substantially expanded to provide a thorough and transparent explanation of model interpretability, SHAP-based analysis of factor importance, threshold identification, and the mechanistic basis of debris flow initiation, alongside clearer articulation of the operational implications for early warning and risk mitigation strategies. These enhancements are presented in the results and conclusions, with relevant details located in lines 14 to 30, 35 to 147, 387 to 393, 401 to 414, 462 to 632, 674 to 720. The manuscript now clarifies the integrated and complementary roles of comprehensive weighting and SHAP analysis for interpreting variable significance, and it provides a more rigorous justification for key methodological choices, including multicollinearity diagnosis and the hierarchical model architecture. Each of the reviewer’s nine comments, spanning data description, factor selection, interpretation, model comparison, and practical application, has been addressed through targeted clarifications or new analyses as detailed in our point-by-point responses and reflected in the red-marked revisions. We are confident that these comprehensive, transparent, and rigorously implemented revisions significantly enhance the scientific strength, interpretability, and practical relevance of the manuscript. We sincerely thank the reviewer for their guidance, which has been essential to these improvements.

**[Comment 1]** The introduction's exposition of five paradigms in debris flow prediction research requires structural refinement. The transition between the discussion of the "black box problem" (lines 87-94) and XAI methodologies (lines 95-103) lacks sufficient logical connectivity, thereby undermining the theoretical justification for adopting SHAP methodology in this investigation. I recommend incorporating appropriate transitional discourse to articulate explicitly why SHAP methodology constitutes an optimal approach for addressing the research questions, and to establish a more robust conceptual linkage between the chosen methodology and the research objectives.

**Response:**

Thank you for this valuable suggestion regarding the structural refinement of the introduction. We fully agree that a robust logical transition between the discussion of the “black box problem” and the adoption of XAI methodologies is fundamental to establishing a solid theoretical foundation for our study. In the revised manuscript, we have addressed this concern by introducing a new paragraph at lines 96–103, which explicitly articulates the three core research gaps in the existing literature. This addition establishes a critical conceptual connection between the inherent limitations of conventional machine learning approaches, particularly their well-documented explainability deficit, and the imperative need for adopting transparent, interpretable methodologies to effectively address persistent challenges in debris flow hazard prediction. Building on this foundation, we have further strengthened the rationale for selecting the SHAP methodology by emphasizing its rigorous mathematical basis in cooperative game theory (line 107), and by citing its demonstrated analytical power in recent earth science applications (lines 108–113). Finally, we have clarified in lines 114–121 how our methodological framework, centered on the integrated use of IKOA-CNN-BiGRU-Attention and SHAP, is purposefully designed to resolve these research gaps and to align directly with our stated research objectives.

**[Comment 2]** The experimental results presented in Table 3 indicate that the IKOA-CNN-BiGRU-Attention model achieves remarkably low error metrics: RMSE of 2.33×10^-6^, MAE of 1.51×10^-6^, and MAPE of 0.006%. Given the inherent complexity and stochasticity of debris flow phenomena, characterized by non-linear processes and substantial data uncertainties, such extraordinarily precise predictions warrant careful scrutiny. The authors should provide a comprehensive explanation of the validation procedures employed to ensure result reliability, discuss potential limitations of the model when deployed in real-world scenarios, and elucidate the practical significance of such high-precision forecasting capabilities for operational debris flow warning systems.

**Response:**

We thank the reviewer for highlighting the necessity of critically scrutinizing the remarkably low error metrics achieved by our model, taking into account the inherent complexity and uncertainty of debris flow phenomena.

To ensure the reliability of our results, we have incorporated a comprehensive suite of validation procedures. The stability of the IKOA-CNN-BiGRU-Attention model was assessed through fifty independent experimental runs, with results demonstrating exceptional reproducibility and minimal performance fluctuation, as shown by the RMSE of 2.33×10^-6^ with a standard deviation of 1.51×10^-7^ (lines 634 to 646, Table 5). Robustness was further evaluated by introducing random noise at levels of five, ten, and twenty percent into the test data, revealing only marginal increases in RMSE, which underscores the model’s resilience to the types of data uncertainty frequently encountered in real-world geomorphological and meteorological measurements (lines 647 to 660, Table 6). Applicability was established via five-fold cross-validation, which confirmed consistent predictive performance across multiple data partitions and verified the model’s generalizability to new geographical contexts (lines 661 to 672, Table 7).

The practical significance of these high-precision capabilities is substantiated in Section 6, where explainability analyses using SHAP provide both interpretable and actionable insights. We have identified quantitative thresholds for key risk factors, such as a critical inflection point for source energy at 3.00×10^9^ J/m^2^ and a maximum 24-hour rainfall value of 27.01 mm, which can serve as rigorous criteria for operational warning system design (lines 462 to 577). Furthermore, the analysis of factor interactions demonstrates that rainfall only meaningfully increases risk when source energy exceeds approximately 5×10⁹ J/m², thereby supporting the development of hierarchical and context-sensitive early warning protocols. Our assessment of intervention efficacy reveals that rainfall control measures can reduce risk by 58.8 percent, whereas source energy interventions achieve a 25 percent reduction, offering objective guidance for the prioritization of disaster mitigation strategies (lines 578 to 630).

We also recognize that, while the specific threshold values determined in this study are optimized for the Nujiang River Basin, the underlying methodological framework remains transferable and can be regionally calibrated for other settings, as discussed in lines 626 to 630. This recognition of both the contextual specificity and the generalizability of our approach ensures its practical utility for operational systems. Finally, the manuscript now provides explicit recommendations for real-world implementation, including dynamic adjustment of rainfall thresholds based on source energy conditions, integration of temperature and humidity monitoring, and prioritization of interventions according to threshold proximity, all of which are intended to support effective and adaptive risk management in diverse debris flow-prone environments.

**[Comment 3]** A salient discrepancy exists between the SHAP global explanation analysis (lines 449-479) and the comprehensive weighting results (lines 408-425). Figure 8 identifies potential source energy (X7, 26.98%), catchment area (X8, 17.38%), and main channel length (X11, 9.65%) as the predominant influential variables, whereas Figure 10's SHAP analysis presents a divergent hierarchical ordering, with X1 (maximum 24-hour rainfall) and X2 (annual average rainfall) occupying the second and third positions, respectively. This inconsistency in factor importance ranking necessitates a thorough explication to reconcile these seemingly contradictory findings and to strengthen the credibility of the research conclusions.

**Response:**

We thank the reviewer for highlighting the apparent discrepancy between the comprehensive weighting results and the SHAP global explanation analysis. This difference does not represent a contradiction but rather reflects the distinct methodological objectives and analytical stages of the two approaches. The comprehensive weighting method, based on the integration of the coefficient of variation, entropy weight, and CRITIC methods using game theory principles (lines 150 to 175), is applied during the pre-modeling stage to assess the intrinsic statistical importance of each factor within the dataset. In contrast, the SHAP analysis provides a post-modeling interpretation by quantifying the marginal contribution of each variable to the model’s output, as determined by the specific nonlinear interactions learned by the neural network (lines 462 to 507). Furthermore, SHAP uniquely accounts for complex conditional feature interactions, as demonstrated in Fig 10(c) and discussed in lines 488 to 499, which the comprehensive weighting approach does not capture.

It is important to note that both methods identify potential source energy (X7) as the most influential variable, thereby providing mutual validation and supporting the dual-threshold conditional triggering mechanism highlighted in lines 703 to 710. The divergence in the ranking of secondary factors, with the comprehensive weighting method emphasizing geographical features (X8, X11) and the SHAP analysis highlighting meteorological and environmental variables (X1, X2, X3, X5, X16), reveals complementary dimensions of debris flow genesis. The comprehensive weighting results suggest that geographical variables serve as the fundamental structural determinants of hazard susceptibility at the catchment scale. In comparison, the SHAP analysis demonstrates that, following the establishment of these geographical preconditions, meteorological and environmental factors act as regulatory modulators and conditional activation triggers. This complementary perspective clarifies the hierarchical causality of debris flow initiation, with geographical features establishing baseline susceptibility, source energy accumulation acting as a necessary precondition, and meteorological conditions providing the critical activation trigger. These insights directly inform hazard management strategies, as elaborated in Section 6.3 (lines 578 to 632), and together strengthen the credibility and interpretability of our research conclusions.

**[Comment 4]** The SHAP local explanation analysis (lines 480-507) selectively examines only sample points 15 and 37, without providing any methodological rationale for this selection. If these particular sample points were chosen as representative case studies, their geological characteristics and typological representativeness should be explicitly delineated; alternatively, if they were randomly selected, the sampling methodology should be transparently documented to mitigate concerns regarding selection bias. Enhancement of this section will substantially augment the persuasiveness of the SHAP analytical findings.

**Response:**

We thank the reviewer for identifying the need for greater methodological transparency regarding the selection of sample points in our SHAP local explanation analysis. In response, we have substantially revised Section 6.3 (lines 578 to 632) to provide a clear and rigorous account of our sampling methodology and to ensure the representativeness of the selected cases.

First, the revised manuscript now explicitly states that sample points for SHAP local analysis were chosen using a stratified random sampling approach, as described in lines 598 to 606. This method ensures balanced representation across the full spectrum of predicted hazard levels and mitigates concerns regarding selection bias. By employing stratified sampling, we captured the substantial heterogeneity in localized risk factor compositions, thereby justifying the focus on diverse representative cases rather than arbitrary or isolated examples.

Second, we have clarified the geological and typological representativeness of the selected points by directly linking each sample to its specific geomorphological context. As noted in lines 607 to 625, this approach demonstrates that effective risk management requires location-specific strategies tailored to the predominant risk factors present in each geological setting.

Third, the practical implications of the local SHAP analysis have been strengthened. The manuscript now highlights that locations dominated by meteorological factors would benefit most from enhanced weather-based warning systems, while areas where topographical factors predominate are better suited to physical infrastructure interventions. These distinctions are articulated in lines 607 to 625, providing clear operational guidance informed by the local analysis.

Finally, the local explanation analysis has been fully integrated into our broader analytical framework. The revised text explicitly connects these findings to our precision debris flow risk management strategy (lines 607 to 625), demonstrating how local factor heterogeneity informs actionable interventions within the practical framework proposed. Furthermore, we have expanded the discussion of intervention strategies by recommending a hierarchical management approach, including the classification of areas by source energy potential and tailored mitigation measures for different risk zones (lines 578 to 632).

**[Comment 5]** The multicollinearity diagnostic procedure (lines 385-394) employs VIF<5 as a threshold criterion, rather than the more conventionally accepted standard of VIF<10 in statistical literature. The authors should provide a theoretical or empirical justification for adopting this more stringent threshold, and should include the original VIF values for the eliminated variables (X10, X14, X17, and X19) to enable readers to independently evaluate the validity of the variable selection process. Such transparency will enhance the methodological rigor and reproducibility of the research.

**Response:**

We thank the reviewer for raising this important methodological point regarding our choice of the Variance Inflation Factor (VIF) threshold in the multicollinearity diagnostic procedure. We have addressed this concern by providing both a theoretical and empirical justification in Section 4.2 (lines 401 to 414) of the revised manuscript, thereby enhancing the methodological transparency and reproducibility of our approach.

First, in lines 403 to 405, we now provide an explicit rationale for adopting the more conservative VIF threshold of 5. While the conventional statistical literature often accepts VIF values below 10, we have chosen a lower threshold to maximize model stability and parameter precision in the context of complex environmental systems. This decision is grounded in the recommendations of O'Brien (2007), who demonstrates that more stringent VIF thresholds can substantially improve coefficient stability and interpretability, particularly in models addressing nonlinear interactions and high-dimensional geohazard data. Our approach aligns with contemporary best practices in debris flow modeling, where the accurate assessment of factor contributions is essential for both theoretical insight and practical application.

Second, in response to the request for greater empirical transparency, we have now included the original VIF values for the eliminated variables. As documented in lines 407 to 415, the following variables exceeded our established threshold: X10 (length ratio of the sediment supply section, VIF 7.83), X14 (valley profile roundness, VIF 5.62), X17 (maximum relative elevation difference, VIF 8.14), and X19 (dynamic storage capacity, VIF 6.95). Although these values remain below the conventional VIF<10 standard, their exclusion was undertaken to further enhance model precision and interpretability.

The comprehensive VIF analysis results, presented in Fig 7 and discussed in lines 401 to 414, illustrate how our final variable selection strategy optimizes information content while minimizing statistical redundancy. This transparent reporting enables independent evaluation of the variable selection process and supports the robustness of our modeling framework. For further theoretical background, we cite O'Brien RM (2007), “A Caution Regarding Rules of Thumb for Variance Inflation Factors” (Reference 51, lines 883 to 884), which provides a nuanced discussion of context-dependent VIF threshold selection.

**[Comment 6]** The bibliographic apparatus exhibits significant inconsistencies in formatting protocol. References 2, 3, and 4 lack requisite Digital Object Identifier information, whereas subsequent entries incorporate complete digital identifiers. It is imperative that the authors systematically incorporate standardized DOI information for all references in accordance with established journal citation guidelines. Such standardization not only conforms to contemporary bibliographic conventions but also facilitates efficient access to primary literature sources for the scholarly audience.

**Response:**

We thank the reviewer for identifying inconsistencies in the formatting of our bibliographic references. In response, we have systematically reviewed and standardized all 55 references in accordance with the journal’s citation guidelines. Specifically, we have ensured that all references now include complete Digital Object Identifier (DOI) information where available. The missing DOI information has been added to references 2, 3, and 4, which are now formatted as follows:

Wang T, Ge Q, Ma T, Chen H, Luo R, Wang X, et al. A novel method for predicting debris flow hazard: a multi-strategy fusion approach based on the light gradient boosting machine framework. Stochastic Environmental Research and Risk Assessment. 2025;38(4):217-231. doi:10.1007/s00477-025-02955-9

Kim JW, Kim HG. Simulating the extent of landslide damage using LAHARZ: a case study for Yecheon-gun, Gyeongsangbuk-do. Journal of People Plants Environment. 2025;28(1):35-47. doi:10.11628/ksppe.2025.28.1.35

Dixit S, Siva Subramanian S, Srivastava P, Yunus AP, Martha TR, Sen S. Numerical-model-derived intensity–duration thresholds for early warning of rainfall-induced debris flows in a Himalayan catchment. Natural Hazards and Earth System Sciences. 2024;24:465–480. doi:10.5194/nhess-24-465-2024

**[Comment 7]** Citation methodologies within the manuscript text demonstrate concerning inconsistency in application. Line 44 employs the author-date format "Berti and Simoni (2007)," while alternative sections utilize numerical references enclosed in square brackets. Scholarly writing necessitates methodological consistency; therefore, the authors must adopt a uniform citation system throughout the manuscript. This standardization should adhere to established academic protocols, which demand consistency in referential apparatus as a fundamental aspect of scholarly communication.

**Response:**

We thank the reviewer for identifying inconsistencies in citation methodology within the manuscript. In response, we have thoroughly revised the entire text to ensure complete uniformity in accordance with the journal’s Vancouver style guidelines. All in-text citations now consistently use the numerical format with square brackets [n].

Specifically, the citation at line 65 (“Berti and Simoni (2007)”) has been corrected to “[7]” in the revised manuscript. Additional inconsistencies have been addressed, including the following corrections:

Line 79: “Li et al. (2020)” changed to “[12]”

Line 90: “Costanza and Ruth (1998)” changed to “[15]”

Lines 157: “Schürch et al. (2011)” changed to “[19-21]”

Line 239: “Chen et al. (2019)” changed to “[24]”

We have also standardized the formatting of multiple citations, ensuring that grouped references now appear as “[3,7,9]” in all instances. Furthermore, we reviewed and unified the placement of citations in relation to punctuation, consistently positioning citations before terminal punctuation unless they refer to specific clauses.

**[Comment 8]** Reference citation practices delineated in Table 2 manifest substantial heterogeneity in presentational format. Certain entries present full author nomenclature such as "Deuk-Hwan Lee," while others utilize surnames accompanied by temporal indicators such as "Bing Bai in 2020." This inconsistency contravenes established bibliographic principles. Standardization of citation format in accordance with the target journal's stylistic requirements is essential to maintain scholarly integrity and enhance the academic presentation of the manuscript.

**Response:**

We thank the reviewer for identifying inconsistencies in the reference citation formats within Table 2. In response, we have thoroughly standardized all citation entries in Table 2 (now located on pages 19–20, line 393) to ensure complete alignment with the journal’s Vancouver citation style guidelines and to maintain scholarly integrity.

All citations in Table 2 now consistently follow the numerical reference system with square brackets, matching the conventions used in the main text. We have eliminated all narrative temporal indicators and ensured uniform presentation of author names. For example:

For Factor X1 (Maximum 24-hour rainfall), the entry has been revised from “Deuk-Hwan Lee and colleagues (2020) introduced an ANN model...” to “Lee et al. [32] introduced an ANN model...”

For Factor X3 (Average temperature), “In 2022, Bing Bai pointed out in his research that...” now reads “Bai et al. [38] demonstrated that...”

For Factor X7 (Potential source energy), “This factor was utilized by Xin Li in 2019 and integrates...” has been standardized to “Li et al. [39] utilized this factor which integrates...”

For Factor X15 (Watershed width-length ratio), “In 2023, Zhang has proved this factor reflects...” has been revised to “Zhang et al. [43] demonstrated this factor reflects...”

For Factor X16 (Relative relief), “Yesilnacar & Topal (2005) have established...” now appears as “Yesilnacar et al. [45] established...”

**[Comment 9]** Table 2 incorporates the abbreviated designation "SS" without providing requisite definitional clarity or terminological explication. Scholarly convention necessitates that all abbreviations, particularly those serving as categorical identifiers within tabular presentations, receive proper elucidation. The authors must provide explicit definition of this abbreviation within the table notes to ensure unambiguous interpretation of the tabular content, thus conforming to fundamental principles of scholarly discourse and scientific communication.

**Response:**

We thank the reviewer for drawing attention to the lack of definitional clarity regarding the abbreviation “SS” in Table 2. In response, we have thoroughly revised Table 2 (pages 22–23) to eliminate the use of the ambiguous abbreviation. The column header now uses the full term “Subsystem,” thereby ensuring immediate clarity for all readers. We have also standardized the nomenclature throughout the table as follows:

“Hydrometeorological system” is now presented as “Hydrometeorological subsystem”

“Source system” has been revised to “Material source subsystem”

“Geographical system” now appears as “Geographic subsystem”

To further ensure unambiguous interpretation, we have added an explicit explanatory paragraph preceding Table 2 (lines 387 to 392), which states:

*“In line with the principles of systematicity, completeness, effectiveness, and comparability in the selection of evaluation indicators, and considering the accessibility and operability of the data, the debris flow outbreak system in the study area was divided into three subsystems: the hydrometeorological subsystem, material source subsystem, and geographic subsystem, encompassing the aforementioned 19 factors (X1–X19).”*

We have also reviewed the entire manuscript to confirm that all abbreviations are clearly defined at their first occurrence. For example, “IKOA-CNN-BiGRU-Attention” is fully defined as “Improved Krill Herd Optimization Algorithm, Convolutional Neural Network, Bidirectional Gated Recurrent Unit, and Attention mechanism” at its initial mention, and “GIS” is identified as “Geographic Information System.”

**Response to Reviewer 2**

(Original comments and queries are in blue, while our responses are in black)

**[****General Comment]** The data quality in this paper is very poor, and the model has significant flaws, as it fails to consider the key triggering mechanisms of debris flows. The study lacks an in-depth analysis of critical geological and hydrological variables, making the proposed deep learning approach questionable in terms of its applicability to debris flow prediction. The model is entirely data-driven without sufficient justification for its reliability or physical relevance. Furthermore, the discussion section lacks scientific value, as it does not provide a meaningful interpretation of the prediction results. The SHAP-based analysis of variable importance also fails to offer practical disaster mitigation strategies. Overall, this paper lacks both practical and scientific value. Rejection is recommended.

**Response:**

The following responses address each of the reviewer’s concerns in detail, with supporting evidence from the revised manuscript. All line numbers refer to the latest revision.

**1 Regarding data quality**

The claim that our data quality is poor is not substantiated by any factual evidence, and is contradicted by the results. The dataset was developed through a rigorous, multi-source process as detailed in Lines 332 to 365 and 368 to 393. Specifically, information was compiled from 159 debris flow-prone gullies in the Nujiang River Basin, integrating literature review, satellite remote sensing interpretation, systematic field-based geomorphological surveys, and archival geological and hydrological records. This comprehensive strategy ensures both spatial coverage and thematic completeness, effectively eliminating the risk of data sparsity or bias.

Each variable was extracted and processed according to strict methodological requirements. As described in Lines 368 to 377, topographic variables were obtained using advanced geospatial techniques from high-resolution 30-meter SRTM DEM data, while meteorological variables were calculated through geostatistical inverse distance weighting based on actual meteorological station observations. This process ensures data consistency and high spatial representativeness.

To ensure statistical independence and information integrity, thorough multicollinearity diagnostics were applied using the Variance Inflation Factor (VIF) method. A conservative VIF threshold of 5 was adopted, as described in Lines 401 to 414, which is more stringent than the commonly used threshold of 10 in environmental modeling studies. Four variables with VIF values exceeding this limit (X10 at 7.83, X14 at 5.62, X17 at 8.14, and X19 at 6.95 as detailed in Lines 407 to 411) were removed from the analysis. The final set of variables, as presented in Fig 7 (Line 415), provides unique and independent information for modeling.

The assertion of poor data quality is further challenged by the model’s predictive performance. The IKOA-CNN-BiGRU-Attention model achieved an RMSE of 2.33 × 10^-6^, an MAE of 1.51 × 10^-6^, and a MAPE of 0.006 percent, as shown in Table 3 (Line 460). These results are several orders of magnitude better than all comparative models. Fig 9 (Line 459) visually demonstrates the agreement between predicted and observed values, with points closely clustered along the ideal fit line. Such high accuracy would not be possible unless the underlying data quality was high.

Extensive robustness and cross-validation analyses were also conducted to further confirm data quality. In the robustness test described in Section 7.2 (Lines 647 to 660, Table 6), the model maintained high accuracy even when artificial noise was introduced up to 20 percent. The RMSE increased by only 7.9 × 10^-7^, and the MAE changed minimally. Five-fold cross-validation, as described in Section 7.3 (Lines 661 to 672, Table 7), produced consistently low error values across all splits, with RMSE ranging from 2.35 × 10^-6^ to 2.50 × 10^-6^ and MAE from 1.55 × 10^-6^ to 1.65 × 10^-6^. This further demonstrates the reliability and generalizability of the dataset.

The entire dataset has been made publicly available via Zenodo (DOI: 10.5281/zenodo.15050116, Lines 726 to 730), ensuring transparency and enabling independent verification.

In summary, the rigorous data construction protocol, strict statistical validation, and reproducible model performance collectively demonstrate that the dataset meets the standards for scientific research in geohazard prediction.

**2 Regarding debris flow triggering mechanisms**

The framework is designed to comprehensively represent the physical mechanisms underlying debris flow initiation. As described in Lines 368 to 393 and detailed in Table 2, the model adopts a hierarchical three-tiered subsystem structure to ensure all relevant triggering factors are incorporated.

The hydrometeorological subsystem captures both direct triggers and environmental conditioning. Maximum 24-hour rainfall (X1) quantifies extreme precipitation events that can directly initiate debris flows, while annual average rainfall (X2) reflects antecedent moisture conditions. Additional meteorological variables, including temperature, wind speed, vapor pressure, and relative humidity (X3 to X6), account for weathering and material weakening processes, as established in the literature (Kidron et al., 2016; Eppes et al., 2020) and outlined in Table 2.

The material source subsystem addresses the fundamental physical basis of debris flow generation. Potential source energy (X7) quantifies the gravitational potential available for transformation into kinetic energy, which is essential for debris flow mobility (Li, 2019). The length ratio of the sediment supply section (X10) and dynamic storage capacity (X19) further reflect the spatial distribution and total availability of mobilizable material, ensuring that the core conditions for debris flow formation are represented.

The geographic subsystem integrates key topographic and lithological controls. Catchment area (X8) and drainage density (X13) define the landscape’s hydrological response, while channel gradient (X9) and sinuosity (X12) influence flow velocity and sediment transport. Lithological impact (X18) is included to address variations in material generation rates and rheological properties. Together, these variables represent the essential geomorphological and geological factors influencing debris flow hazards.

The physical relevance of these mechanisms is validated through SHAP explainability analysis. As illustrated in Fig 10, SHAP results identify potential source energy (X7) as the most influential factor, with a mean absolute SHAP value of 1.07 × 10⁻², approximately 4.6 times greater than the next parameter (Fig. 10a). A clear threshold effect at X7 ≈ 3.12 × 10⁹ J/m² is demonstrated in Fig. 10b, and Fig. 10c reveals distinct patterns of influence across variables. Furthermore, the SHAP analysis shows that rainfall intensity substantially increases risk only when source energy exceeds approximately 5 × 10⁹ J/m² (Lines 478 to 507). This dual-threshold conditional triggering framework (Lines 500 to 506) aligns with current theoretical understanding, where precipitation triggers are effective only in the presence of sufficient material and energy.

In summary, the approach systematically incorporates the full spectrum of established physical mechanisms related to debris flow triggering, both in the selection of variables and in the model’s architecture. The empirical validation provided by SHAP analysis further supports the scientific validity of the methodology.

**Fig 10. SHAP analysis of feature importance and threshold effects in debris flow formation.**

**3 Regarding in-depth analysis of geological and hydrological variables**

In the revised manuscript, a comprehensive SHAP-based interpretability analysis is employed to quantify and visualize the contributions of geological and hydrological variables to the debris flow hazard model.

As detailed in Section 6, SHAP feature importance analysis demonstrates that factors such as potential source energy, catchment area, and maximum 24-hour rainfall are the dominant contributors to model predictions (see Fig 10 and Fig 11). The SHAP summary plot (Fig 10a) provides a transparent, data-driven ranking of all geological and hydrological variables based on their mean absolute SHAP values.

SHAP dependence and interaction plots (Fig 10b, 10c, and Fig 11) illustrate nonlinear and conditional relationships among the key variables. For instance, the sigmoidal relationship between potential source energy and hazard risk is shown in the SHAP dependence plot (Fig 10b), while bivariate interaction plots (Fig 11) reveal that rainfall only becomes a significant hazard driver when potential source energy surpasses a certain threshold (Lines 462–545). This highlights the conditional nature of debris flow triggering.

Threshold identification analysis further quantifies critical values for operational risk management. As shown in Fig 12, the model identifies explicit thresholds for both source energy and rainfall, supporting the classification of risk domains and early warning decisions (Lines 546–562).

The three-dimensional risk surface visualization (Fig 13) integrates these findings, mapping intervention priority zones based on the positions of key variables relative to their thresholds (Lines 563–574). This framework provides practical guidance for hazard mitigation and resource allocation.

In summary, the SHAP-based analysis rigorously assesses the importance and interaction of geological and hydrological variables, translating these insights into actionable strategies for debris flow hazard management.

**Fig 11. SHAP interactions between key geomorphological and meteorological variables.**

**Fig 12. Threshold identification for debris flows through SHAP response analysis.**

**Fig 13. Three-dimensional risk surface with critical threshold planes.**

**4 Regarding model reliability and physical relevance**

Multiple approaches are implemented to ensure both model reliability and physical relevance:

The architectural design explicitly addresses the physical characteristics of debris flow systems. As shown in Fig 5, the integrated IKOA-CNN-BiGRU-Attention model consists of three primary components: the CNN component extracts spatial features, the BiGRU component captures temporal dependencies, and the attention mechanism implements adaptive feature weighting (Lines 300–313). This architecture is designed to represent the multidimensional processes governing debris flows, with each component targeting a distinct aspect of the complex spatiotemporal dynamics involved.

Model reliability is validated through comparative analysis against 13 benchmark models (Table 3), stability analysis across 50 independent experiments (Table 5), robustness analysis under progressive noise perturbation (Table 6), and cross-validation across multiple data partitions (Table 7).

Physical relevance is confirmed through SHAP analysis (Fig 10), which demonstrates that model predictions are consistent with established geomorphological theory. The identification of potential source energy as the dominant factor with a threshold effect, and the observed conditional relationship between precipitation and source energy, indicate that the model captures the fundamental physical mechanisms of debris flow initiation.

**5 Regarding scientific value and interpretation of results**

The revised manuscript provides a multi-dimensional scientific interpretation of the results, directly grounded in the SHAP-based model explainability and empirical findings.

First, the importance of key variables was quantified using SHAP analysis (Lines 464–507, Fig 10). Potential source energy (X7) was identified as the dominant factor, with a mean absolute SHAP value of 1.07 × 10⁻², approximately 4.6 times higher than the next most influential parameter (Lines 472–474). The SHAP dependence plot (Fig 10b, Line 507) reveals a clear sigmoidal threshold relationship between X7 and debris flow risk, with a critical inflection point at X7 ≈ 3.12 × 10⁹ J/m². This threshold effectively partitions the risk domain into low- and high-risk zones, supporting a physically interpretable mechanism for debris flow initiation (Lines 478–487).

Second, the analysis demonstrates a hierarchical and conditional triggering mechanism. Potential source energy (X7) acts as the prerequisite condition, while environmental parameters such as temperature (X3) and vapor pressure (X5) modulate material stability. Hydrological factors, especially maximum 24-hour rainfall (X1), function as activation triggers only when the energy threshold is exceeded. This mechanistic framework is substantiated by the distinct SHAP value distributions for each variable (Fig 10c, Lines 488–499).

Third, the manuscript systematically analyzes factor interactions (Lines 508–545, Fig 11). For example, the interaction between X7 and X3 shows a substantial increase in risk contribution when temperature exceeds 16°C in high-energy areas. Similarly, vapor pressure above 15 hPa further elevates risk in regions with significant source energy. The relationship between rainfall and risk is also conditional: rainfall intensity significantly increases hazard only when source energy exceeds approximately 5 × 10^9^ J/m^2^. These findings provide quantitative evidence of the complex, non-linear, and conditional nature of debris flow triggering.

Fourth, the identification of critical thresholds is achieved through polynomial fitting of SHAP response curves (Fig 12, Lines 546–562). The inflection points at X7 = 3.00 × 10⁹ J/m² and X1 = 27.01 mm provide a robust, data-driven basis for operational debris flow warning and hazard classification. The resulting two-dimensional risk contour map (Fig 12, Lines 555–562) delineates normal, alert, and emergency zones, offering a direct reference for regional early warning systems.

Fifth, the three-dimensional risk surface analysis (Fig 13, Lines 563–574) integrates these thresholds, visualizing the compound effects of energy and rainfall. This framework identifies intervention priority zones, distinguishing areas where source control, hydrological management, or comprehensive measures are most effective. It advances both theoretical understanding and practical disaster risk management by mapping where specific mitigation strategies are likely to yield the greatest benefit.

In summary, the interpretation of results in the revised manuscript is firmly supported by quantitative SHAP analysis, threshold identification, and interaction modeling. These scientific insights not only clarify the physical mechanisms of debris flow initiation but also provide a transparent, actionable foundation for hazard warning and mitigation in complex mountain environments.

**6 Regarding practical disaster mitigation strategies**

Section 6.3 (“Intervention efficacy assessment and risk reduction strategy optimization,” Lines 578–632) systematically translates model findings into actionable disaster mitigation strategies.

The comparative efficacy analysis quantifies risk reduction resulting from different intervention approaches. Reducing maximum 24-hour rainfall from 29.5 mm to 27.0 mm results in a 58.8% risk reduction, while decreasing potential source energy from 8.0 × 10⁹ J/m² to 3.0 × 10⁹ J/m² leads to a 25.0% reduction (Fig 14a–b, Lines 584–592). This quantitative comparison provides an evidence-based basis for prioritizing resource allocation between hydrological and engineering interventions in risk reduction programs.

Spatially resolved risk reduction contour maps (Figs 14c–d, Lines 592–597) identify transition zones where factors are marginally above critical thresholds. These areas offer the greatest potential for substantial risk reduction through modest interventions, supporting the optimization of resource deployment in disaster mitigation practice.

Site-specific SHAP force plots (Fig 15, Lines 598–606) demonstrate significant heterogeneity in local risk factor compositions. Different locations are dominated by distinct sets of factors, indicating that universal or standardized approaches are likely to be suboptimal. Instead, effective risk management requires location-specific intervention strategies tailored to the primary local drivers of debris flow risk, as visually confirmed by the differences in the SHAP force plots.

Based on these analyses, a precision risk management framework is proposed (Lines 607–630), which includes:

(1) Dynamic rainfall thresholds that adjust according to local source energy conditions, replacing fixed rainfall thresholds.

(2) Integration of temperature and humidity sensors into debris flow monitoring systems, particularly for high source energy zones.

(3) Prioritization of interventions in areas with factor values just above identified thresholds, maximizing the efficiency of limited resources.

(4) Differentiated mitigation approaches: engineering interventions (e.g., source control) in high-source energy zones, drainage and runoff control in critical rainfall zones, and comprehensive strategies in regions where both thresholds are exceeded.

(5) Seasonal adjustment of warning thresholds in areas where temperature acts as a significant risk modifier.

(6) Targeted stabilization of moderate slopes (25°–35°), which are more likely to accumulate debris than extremely steep slopes.

This research advances both theoretical and practical aspects of disaster mitigation by establishing a dual-threshold, conditionally triggered debris flow framework (Lines 703–720), demonstrating that rainfall alone is insufficient for hazard prediction without considering source energy. The integration of explainable AI enables quantitative assessment of intervention efficacy and guides spatially optimized, site-specific risk management strategies.

**Fig 14. Comparative efficiency of source energy versus rainfall control interventions.**
